# Astragalus Polysaccharide Suppresses Inflammation and Promotes Apoptosis in Hypertrophic Scars by Suppressing OGT-Mediated Nrf2 O-GlcNAcylation

**DOI:** 10.5812/ijpr-168614

**Published:** 2026-04-18

**Authors:** Hengfei Wang

**Affiliations:** 1 Medical College of Hexi University, Zhangye, China

**Keywords:** Hypertrophic Scar, Astragalus Polysaccharides, O-GlcNAcylation, Nrf2

## Abstract

**Background:**

Hypertrophic scars (HS) arise from excessive tissue proliferation during wound healing, with Nrf2 involved, though the underlying mechanism remains unclear. Astragalus polysaccharides (APS) have anti-inflammatory and antioxidant properties, but their therapeutic effects and mechanisms in HS remain unreported.

**Objectives:**

This study intends to clarify how APS target protein O-GlcNAcylation to treat HS.

**Methods:**

A HS mouse model was established by subcutaneous injection of bleomycin (BLM) in C57BL/6 mice. Histopathology (H&E and Masson staining), ELISA, CCK-8, flow cytometry, western blot, and co-immunoprecipitation were performed to assess pathological changes, cell viability, apoptosis, inflammatory cytokine levels, and protein O-GlcNAcylation.

**Results:**

Astragalus polysaccharides treatment significantly inhibited scar formation and reduced inflammatory cytokine levels in HS mice. In human hypertrophic scar fibroblasts (HHSFs), APS suppressed cell viability and inflammation while promoting apoptosis. Mechanistically, APS decreased global O-GlcNAcylation levels and downregulated the protein expression of OGT and Nrf2. Mechanistically, OGT interacted with Nrf2, enhancing its stability via O-GlcNAcylation at S199. Moreover, Nrf2 overexpression reversed APS-induced changes in HHSF viability, inflammation, and apoptosis.

**Conclusions:**

This study identifies the OGT-mediated O-GlcNAcylation of Nrf2 as a novel regulatory mechanism in HS progression. By suppressing this axis, APS demonstrates therapeutic potential for HS. These findings highlight O-GlcNAcylation as a promising therapeutic target and support the clinical development of APS for fibrotic skin disorders.

## 1. Background

Introduction Hypertrophic scars (HS) are abnormal fibrous tissue proliferations that develop during aberrant wound healing, characterized by excessive collagen deposition and raised, firm lesions confined to the original injury site (1, 2). Clinically, HS presents as erythematous, pruritic, and painful lesions, often causing functional limitations in joint areas (3). Hypertrophic scars commonly arise following traumatic injuries, burns, surgeries, or improper wound closure, with risk factors including infection, tension on the wound edges, and delayed healing (4, 5). While not life-threatening, HS can lead to significant functional impairments and psychological distress due to their disfiguring appearance. Recent studies have explored the molecular mechanisms underlying HS, with growing attention to the role of Nrf2, a key regulator of cellular antioxidant and anti-inflammatory responses. In wound healing, Nrf2 promotes tissue repair by activating antioxidant enzymes such as HO-1 to counteract oxidative stress and inflammation (6, 7). Furthermore, evidence shows that Nrf2 is abnormally overexpressed in human hypertrophic scar fibroblasts (HHSFs) but downregulated by mesenchymal stem cell treatment, implying its critical role in HS pathogenesis (8). Therefore, investigating the roles and underlying mechanisms of Nrf2 in HS is vital for developing targeted therapies. Astragalus polysaccharides (APS), the primary bioactive components extracted from the traditional medicinal herb Astragalus membranaceus, exhibit diverse pharmacological properties, including immunomodulation, anti-inflammatory, anti-tumor, hypotensive, and antioxidant activities (9, 10). Due to their potent anti-inflammatory properties, APS have been widely applied in the treatment of inflammatory diseases such as atherosclerosis and colitis (11, 12). Inflammation plays a critical role in the progression of HS, and modulation of the inflammatory response has been shown to alleviate HS development (13). Emerging evidence suggests that APS exert their anti-inflammatory effects through the suppression of Nrf2 signaling activation, thereby ameliorating the pathogenesis of various diseases (14, 15). However, whether APS can effectively target HS remains to be elucidated. Moreover, it is currently unclear whether APS regulate Nrf2 expression specifically in the context of HS formation. O-GlcNAcylation is a dynamic post-translational modification in which N-acetylglucosamine (GlcNAc) is covalently attached to serine or threonine residues of target proteins (16). This reversible process is tightly regulated by O-GlcNAc transferase (OGT) and O-GlcNAcase (OGA), enzymes that mediate GlcNAc addition and removal, respectively (17). O-GlcNAcylation plays critical roles in cellular signaling, metabolic stress responses, and inflammation by modulating protein function, stability, and subcellular localization (18). It has been extensively studied in diseases such as cancer, neurodegeneration, diabetes, and cardiovascular disorders, where dysregulation of O-GlcNAcylation is linked to pathological progression (19-21). Despite these insights, the role of O-GlcNAcylation in HS formation remains poorly understood.

## 2. Objectives

In this study, we explored the therapeutic effects of APS on HS through in vivo and in vitro experiments and investigated the regulation of APS on O-GlcNAcylation and Nrf2 in HS. This study aims to elucidate the underlying mechanisms of APS in treating HS, which may provide new insights into the molecular mechanisms of HS formation and offer potential therapeutic targets for clinical HS management.

## 3. Methods

### 3.1. Animal Study

A total of 30 C57BL/6 mice (male, 8-week-old, 20 - 22 g) were bought from Cyagen Biosciences Co., Ltd. (Suzhou, China). All mice were housed in a specific pathogen-free (SPF) facility under controlled environmental conditions: Temperature at 22 ± 2°C, humidity at 55 ± 10%, and a 12-hour light/12-hour dark cycle, with ad libitum access to food and water. Following a one-week acclimatization period, experiments were initiated. Throughout the study, mice were monitored daily for mental status, food intake, and body weight changes to ensure the absence of disease or abnormal reactions. Mice were randomly divided into five groups (n = 6): The control group, the bleomycin (BLM) group, the BLM+APS (10 mg/kg) group, the BLM+APS (50 mg/kg) group, and the BLM+APS (100 mg/kg) group. Mice in the BLM and all BLM+APS groups underwent HS induction. The BLM-induced skin fibrosis mouse model is commonly used to study the mechanisms underlying HS. In this study, BLM was administered as previously described (22). Mouse dorsal hair was removed using depilatory cream over an area of approximately 3 cm2. Subsequently, 100 μL of BLM solution (0.1 mg/mL, dissolved in PBS) was injected into the depilated area daily for 21 consecutive days. Control mice received an identical treatment with saline instead of BLM. To evaluate the therapeutic effect of APS on the HS mouse model, APS (purity ≥ 98%; Yuanye Bio-Technology, Shanghai, China) was dissolved in sterile saline to prepare stock solutions at concentrations of 1, 5, and 10 mg/mL. The pH of the APS solution was adjusted to 7.4 using 0.1 M NaOH or HCl as needed. Mice in the BLM+APS groups were administered APS via intraperitoneal injection at doses of 10, 50, or 100 mg/kg body weight, corresponding to injection volumes of 10 mL/kg body weight (e.g., 200 μL for a 20 g mouse), daily for 28 days following the completion of BLM induction. Mice in the control and BLM groups received intraperitoneal injections of an equal volume of sterile saline. At the end of the treatment period, all animals were euthanized by inhalation of 5% isoflurane. Skin tissues were collected and fixed in 4% paraformaldehyde, while blood was drawn from the tail vein, centrifuged to obtain serum, and stored at -80 °C for further analysis.

### 3.2. Hematoxylin and Eosin Staining

 Hematoxylin and eosin staining was conducted using a HE staining kit (Beyotime, Shanghai, China). Skin tissues fixed in 4% paraformaldehyde were dehydrated through graded ethanol, cleared in xylene, embedded in paraffin, and made into 5-μm-thick sections. The sections were stained with hematoxylin for 10 min and eosin for 2 min, respectively. Sections were then dehydrated, cleared, and mounted with coverslips, and images were captured using a microscope.

### 3.3. Masson Staining

Masson staining was conducted using a Masson staining kit (Beyotime). The paraffin sections after dewaxing and rehydration were stained with hematoxylin for 5 min, ponceau S-acid fuchsin for 10 min, and light green for 1 min, respectively. The results were observed under a microscope.

### 3.4. Cell Culture and Treatment

Human hypertrophic scar fibroblasts (SUNNCELL, Wuhan, China) and human skin fibroblasts (HSFs; STEM RECELL; Shanghai, China) were cultured in Dulbecco’s Modified Eagle Medium (DMEM; Gibco, Grand Island, NY, USA) supplemented with 10% FBS (Gibco) and 1% penicillin/streptomycin in a humidified 5% CO_2_ incubator at 37°C. HSFs were defined as the normal group, while HHSFs were designated as the HS group. To evaluate the effect of APS, HHSFs were treated with 200 μg/mL APS for 24 h.

### 3.5. Cell Transfection

Empty vector (pcDNA3.1), OGT1 overexpression plasmid (pcDNA3.1-OGT1), and Nrf2 overexpression plasmids (pcDNA3.1-Nrf2) were synthesized by GeneScript (Nanjing, China). Transfection was performed using Lipofectamine 2000 (Invitrogen, Carlsbad, CA, USA) according to the manufacturer’s protocol when cells reached 70% confluence. Following 48 h of transfection, cells were harvested for subsequent analyses (23).

### 3.6. Enzyme-Linked Immunosorbent Assay

The levels of IL-1β, IL-6, and TNF-α in mouse sera, HHSFs, and HSFs were measured using ELISA kits (Beyotime) according to the manufacturer’s protocol. Briefly, 96-well plates were coated with capture antibody, blocked with BSA, and incubated with samples or standards, detection antibody, and HRP-conjugated secondary antibody sequentially, with PBST washes between steps. TMB substrate was added for color development, the reaction was stopped, and absorbance was measured at 450 nm using the microplate reader Synergy H1 (BioTek, Vermont, USA). Concentrations were determined using a standard curve.

### 3.7. Cell Viability

Cell viability was assessed using the CCK-8 Kit (Yeasen, Shanghai, China) according to the manufacturer’s protocol. Briefly, cells were seeded into 96-well plates at a density of 1 × 10^4^ cells per well and cultured overnight to allow attachment. Next, 10 μL of CCK-8 solution was added to each well, and the plates were incubated at 37°C for 2 h. The absorbance at 450 nm was measured using a microplate reader.

### 3.8. Detection of Apoptosis

Apoptosis was detected using flow cytometry with an Annexin V-FITC Apoptosis Detection Kit (Beyotime). Cells were collected by centrifugation, washed with PBS, and resuspended in 195 µL Annexin V-FITC binding buffer. Then, 5 µL Annexin V-FITC and 10 µL propidium iodide (PI) staining solution were added, followed by gentle mixing and incubation at room temperature in the dark for 10 min. After incubation, cells were placed on ice and analyzed immediately using the flow cytometer FACSCanto II (BD Biosciences, Franklin Lake, NJ, USA).

### 3.9. Western Blot

The protein samples were isolated from fibroblasts using RIPA buffer (Beyotime) supplemented with protease and phosphatase inhibitors. The protein concentration was determined using a BCA protein assay kit (Beyotime). Proteins were separated by SDS-PAGE and transferred to PVDF membranes. Membranes were blocked with 5% non-fat milk in TBST for 1 h at room temperature and then incubated with anti-OGT (1:1000, ab96718, Abcam, Cambridge, MA, USA), anti-OGA (1:1000, ab124807, Abcam), anti-Nrf2 (1:1000, ab62352, Abcam), anti-O-GlcNAcylation (1:1000, MA1-072, Thermo Scientific), or anti-β-actin (1:5000, ab8227, Abcam) overnight at 4°C. Afterwards, membranes were incubated with HRP-conjugated secondary antibodies (1:10000, ab6721, Abcam) for 1 h at room temperature. Protein bands were detected using an ECL substrate (Beyotime), and images were captured using a chemiluminescence imaging system.

### 3.10. Co-immunoprecipitation and Immunoprecipitation

The interaction between OGT and Nrf2 was assessed using a Co-IP kit (Beyotime). Fibroblasts were lysed in IP lysis buffer (Beyotime) containing protease inhibitors. The cell lysates were centrifuged at 12000 × g for 5 min at 4°C to obtain the supernatant. Protein A/G magnetic beads (Beyotime) were pre-coated with anti-OGT, anti-Nrf2, anti-IgG, or anti-O-GlcNAcylation for 1 h at room temperature. The supernatant was then incubated with antibody-conjugated beads overnight at 4°C. Afterwards, beads were harvested using a magnetic frame and washed three times with lysis buffer. The interaction between OGT and Nrf2 was determined by western blot analysis.

### 3.11. Immunofluorescence Staining

Fibroblasts were fixed with 0.5 mL of fixative for 10 min, followed by removal of the fixative via centrifugation. The cells were then washed three times with washing buffer, and the cell suspension was applied to glass slides and incubated with blocking solution for 1 h at room temperature. Afterwards, the cells were incubated with anti-OGT or anti-Nrf2 for 1 h, followed by washing the slides with PBS and incubation with HRP-conjugated secondary antibody in the dark for 1 h at room temperature. Finally, the cells were washed three additional times with PBS, counterstained with DAPI for 5 min, rinsed, mounted, and examined under a fluorescence microscope.

### 3.12. Prediction of Nrf2 O-GlcNAcylation sites

The potential O-GlcNAcylation sites in human Nrf2 were predicted using the DictyOGlyc 1.1 server (https://services.healthtech.dtu.dk/services/DictyOGlyc-1.1/), a web-based tool for the prediction of mucin-type O-glycosylation sites in proteins. The full-length amino acid sequence of human Nrf2 was retrieved from the UniProt database (https://www.uniprot.org/). The sequence was submitted to the DictyOGlyc 1.1 server with default parameters (threshold value set to 0.5) to identify candidate O-GlcNAcylation sites. The output results, including the position of each predicted site and its corresponding glycosylation potential score, were visualized and analyzed to select the Ser199 residue for subsequent experimental validation.

### 3.13. Protein Stability Assay

To evaluate the stability of Nrf2 in fibroblasts from healthy people, the cells were treated with 10 μM cycloheximide (CHX; MedChemExpress, Monmouth Junction, NJ, USA), and the protein levels of Nrf2 were measured using western blot at 0, 4, 8, 16, and 24 h following CHX treatment (24).

### 3.14. Quantitative Real-time PCR

Total RNA from HHSFs was isolated using Trizol reagent (Thermo Scientific). RNA was reverse transcribed into cDNA using a reverse transcription kit (Vazyme, Nanjing, China). qPCR was performed on QuantStudio 7 real-time PCR System (Applied Biosystems, CA, USA) with SYBR Green master mix (Vazyme). Relative gene expression levels were calculated using the 2^-ΔΔCt^ method and normalized to β-actin. The primer sequences of Nrf2 are presented below: 5’-TCAGCGACGGAAAGAGTATGA-3’ (sense) and 5’-CCACTGGTTTCTGACTGGATGT-3’ (antisense).

### 3.15. Statistical Analysis

All data were analyzed using SPSS 22.0 software. All data are presented as mean ± SD/SEM from at least three independent biological replicates. Comparisons between two or multiple groups were conducted using Student’s *t*-test or one-way ANOVA. P < 0.05 indicates that the difference is statistically significant.

## 4. Result

### 4.1. Astragalus Polysaccharides Inhibits Skin Fibrosis, Inflammation, and OGT-Mediated O-GlcNAcylation in the Hypertrophic Scars Mouse Model

**Figure 1.**
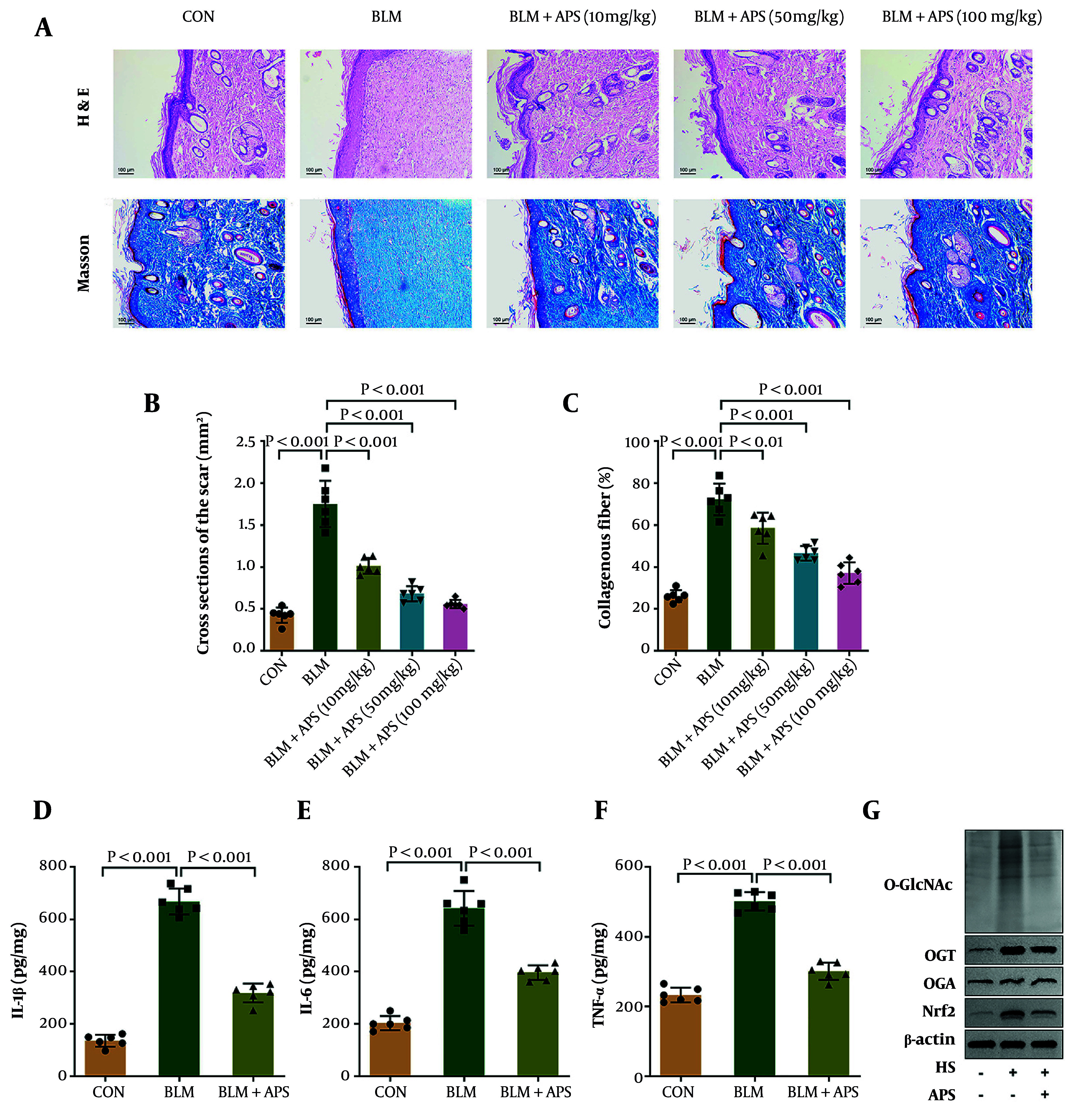
Astragalus polysaccharides (APS) inhibited skin fibrosis, inflammation, and OGT-mediated O-GlcNAcylation in the HS mouse model. A, the pathological changes and collagen deposition of scar tissues were evaluated by HE (hematoxylin and eosin) and Masson (Masson’s trichrome) staining. B and C, the quantification results of HE and Masson staining. D-F, the levels of IL-1β (interleukin-1β), IL-6 (interleukin-6), and TNF-α (tumor necrosis factor-α) in mouse sera were measured by ELISA. G, O-GlcNAcylation and the protein levels of OGT, OGA, and Nrf2 were detected by western blot. CON, control group; BLM, bleomycin-induced HS model group; n = 6 independent biological replicates; P < 0.05 was considered statistically significant, and P values are indicated in the Figure.

To clarify the therapeutic effect of APS on HS in vivo, we established a HS mouse model via BLM induction and investigated the therapeutic efficacy of different APS doses (10, 50, and 100 mg/kg). Compared with the control group, BLM-treated mice exhibited dermal thickening and scar tissue formation. In contrast, APS administration alleviated these pathological changes in a dose-dependent manner, with skin architecture progressively approaching normalcy. Masson staining revealed extensive collagen deposition with dense arrangement in the skin of BLM-induced mice. However, collagen deposition and fiber density in the BLM+APS groups decreased progressively with increasing APS doses (Figure 1A-C). Among the three tested doses, APS at 100 mg/kg demonstrated the optimal therapeutic effect against BLM-induced scarring; therefore, this dose was selected for subsequent investigations. ELISA results showed that the levels of pro-inflammatory cytokines IL-1β, IL-6, and TNF-α were significantly increased in the BLM group and reduced following APS treatment (Figure 1D-F). Western blot results demonstrated that APS decreased the levels of O-GlcNAcylation and OGT protein, as well as downregulated Nrf2 protein in the HS mouse model (Figure 1G). To sum up, these findings demonstrated that APS inhibited the levels of skin fibrosis and inflammation, and OGT-mediated O-GlcNAcylation in the HS mouse model. Additionally, HSFs were treated with 100 mg/kg APS to evaluate its toxicity and specificity. Results showed that APS had no significant effect on HSF viability, apoptosis, or pro-inflammatory cytokine release, indicating that APS is non-toxic to HSFs and exerts its effects in a cell type-specific manner (Figure S1A-F).

### 4.2. Astragalus Polysaccharides Suppresses Inflammation and Cell Viability but Promotes Apoptosis in Human Hypertrophic Scar Fibroblasts with Hypertrophic Scars by Inhibiting OGT-Mediated O-GlcNAcylation

**Figure 2.**
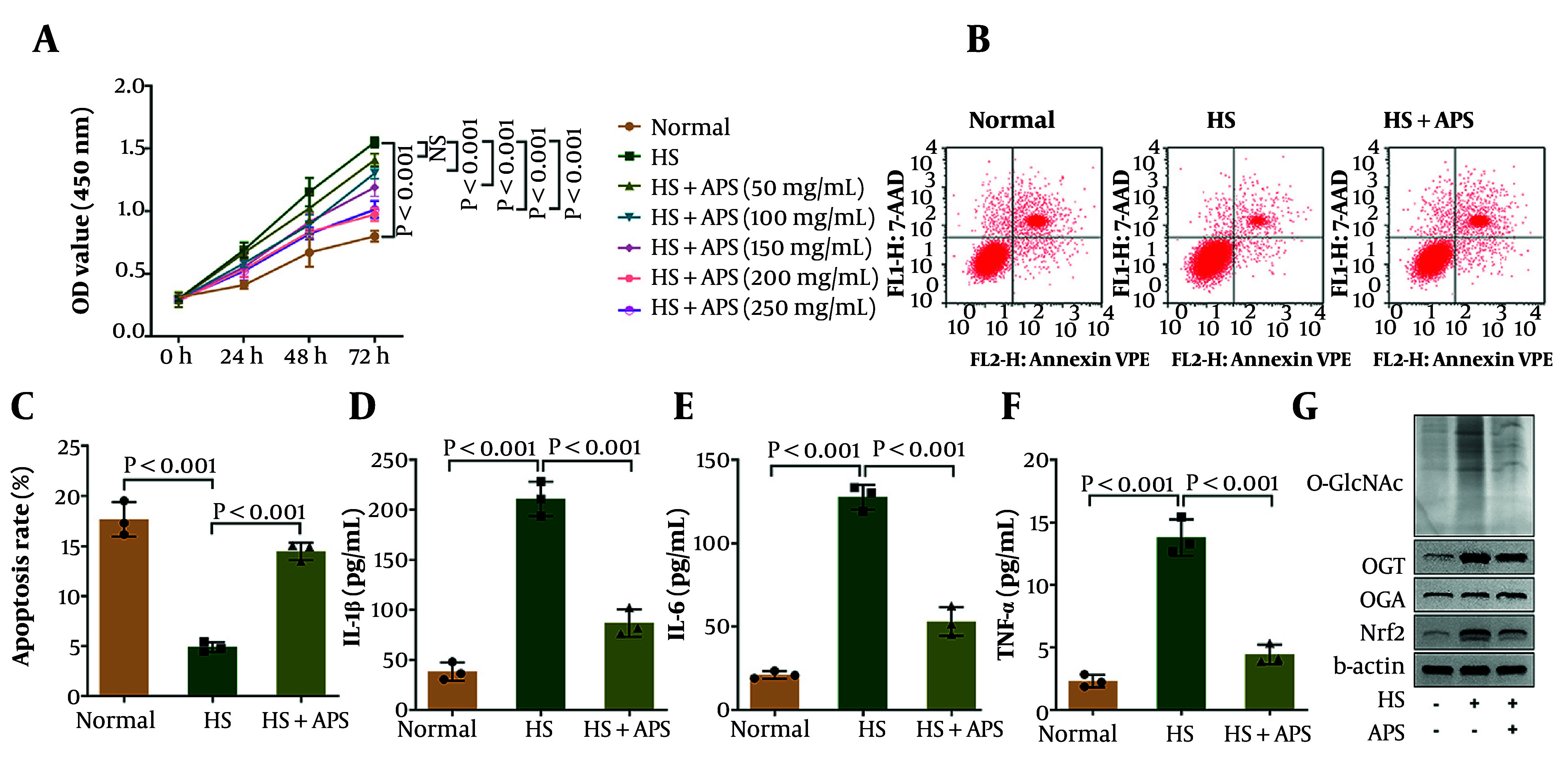
Astragalus polysaccharides (APS) suppressed inflammation and cell viability but promoted apoptosis in human hypertrophic scar fibroblasts (HHSFs) with HS by inhibiting OGT-mediated O-GlcNAcylation. A, cell viability of HHSFs (human hypertrophic scar fibroblasts) and HSFs (human skin fibroblasts) was detected using a CCK-8 kit. B and C, apoptosis of HHSFs and HSFs was evaluated by flow cytometry; D-F, the levels of IL-1β, IL-6, and TNF-α in HHSFs and HSFs were measured by ELISA. G, O-GlcNAcylation and the protein levels of OGT, OGA, and Nrf2 were detected by western blot. n = 3 independent biological replicates; P < 0.05 was considered statistically significant.

Subsequently, we evaluated the therapeutic effect of APS on HHSFs and determined the optimal treatment concentration. Compared with the normal group, cell viability was significantly elevated in the HS group; however, APS treatment reduced viability in a dose-dependent manner (Figure 2A). Given that APS concentrations of 200 and 250 μg/mL yielded comparable improvements in cell viability within the HS group, indicating that 200 μg/mL had already achieved a robust therapeutic effect, this concentration was selected for all subsequent experiments. Flow cytometry assay demonstrated that the apoptosis rate was decreased in the HS group, which was markedly elevated following APS treatment (Figure 2B and C). Additionally, the elevated levels of pro-inflammatory cytokines IL-1β, IL-6, and TNF-α in the HS group were reduced following APS treatment (Figure 2D-F). To investigate whether APS inhibits the development of HS through O-GlcNAcylation, we examined the effects of APS treatment on O-GlcNAcylation and related proteins. Results suggested that the levels of O-GlcNAcylation and OGT protein expression were upregulated in the HS group, whereas APS treatment significantly decreased both parameters. Notably, there was no difference in the protein level of OGA among groups. Moreover, the upregulated protein level of Nrf2 in the HS group was decreased following APS treatment (Figure 2G). In conclusion, these results demonstrate that APS suppressed inflammation and cell viability but enhanced apoptosis in HHSFs by inhibiting OGT-mediated O-GlcNAcylation.

### 4.3. OGT Overexpression Enhances the Stability of Nrf2 Protein by Increasing O-GlcNAcylation at the S199 site of Nrf2

**Figure 3.**
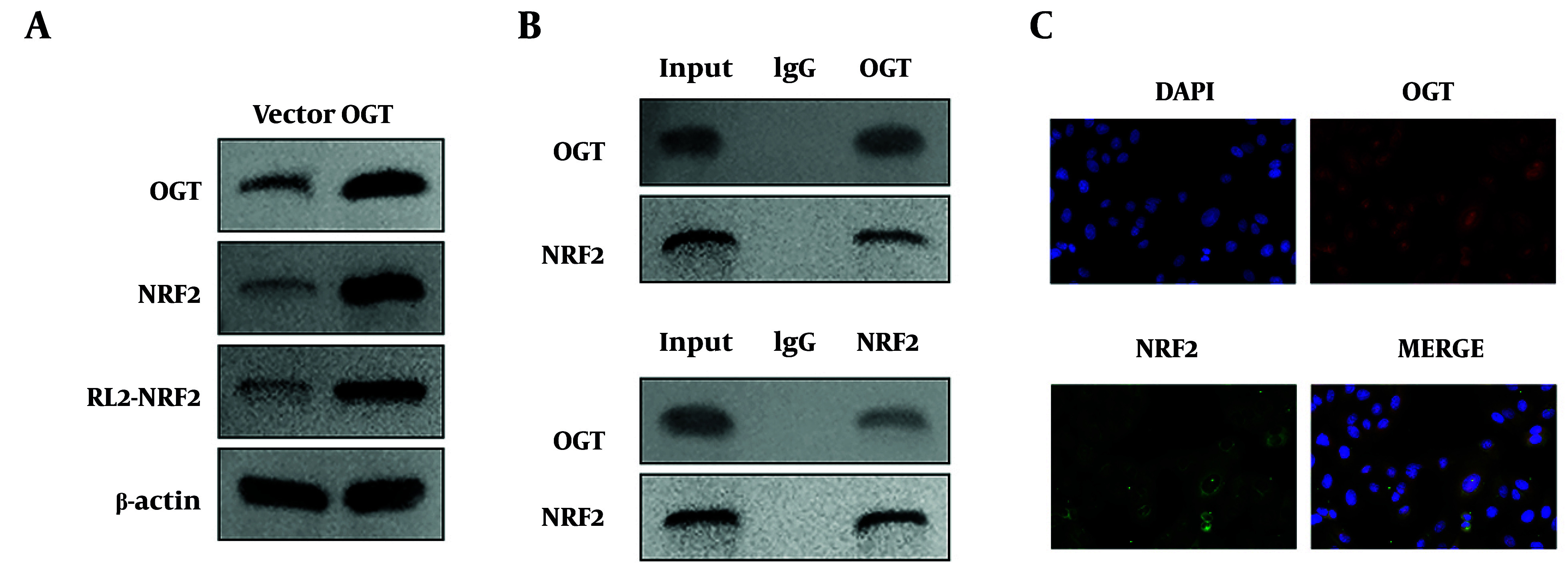
Identification of the interaction between OGT and Nrf2. A, the protein levels of OGT and Nrf2 and the O-GlcNAcylation level of Nrf2 were detected by western blot. B, the interaction between OGT and Nrf2 was evaluated by Co-IP (co-immunoprecipitation); IgG, immunoglobulin G, used as the negative control for Co-IP. C, IF (immunofluorescence) staining was performed to detect the co-localization between OGT and Nrf2. n = 3 independent biological replicates.

**Figure 4.**
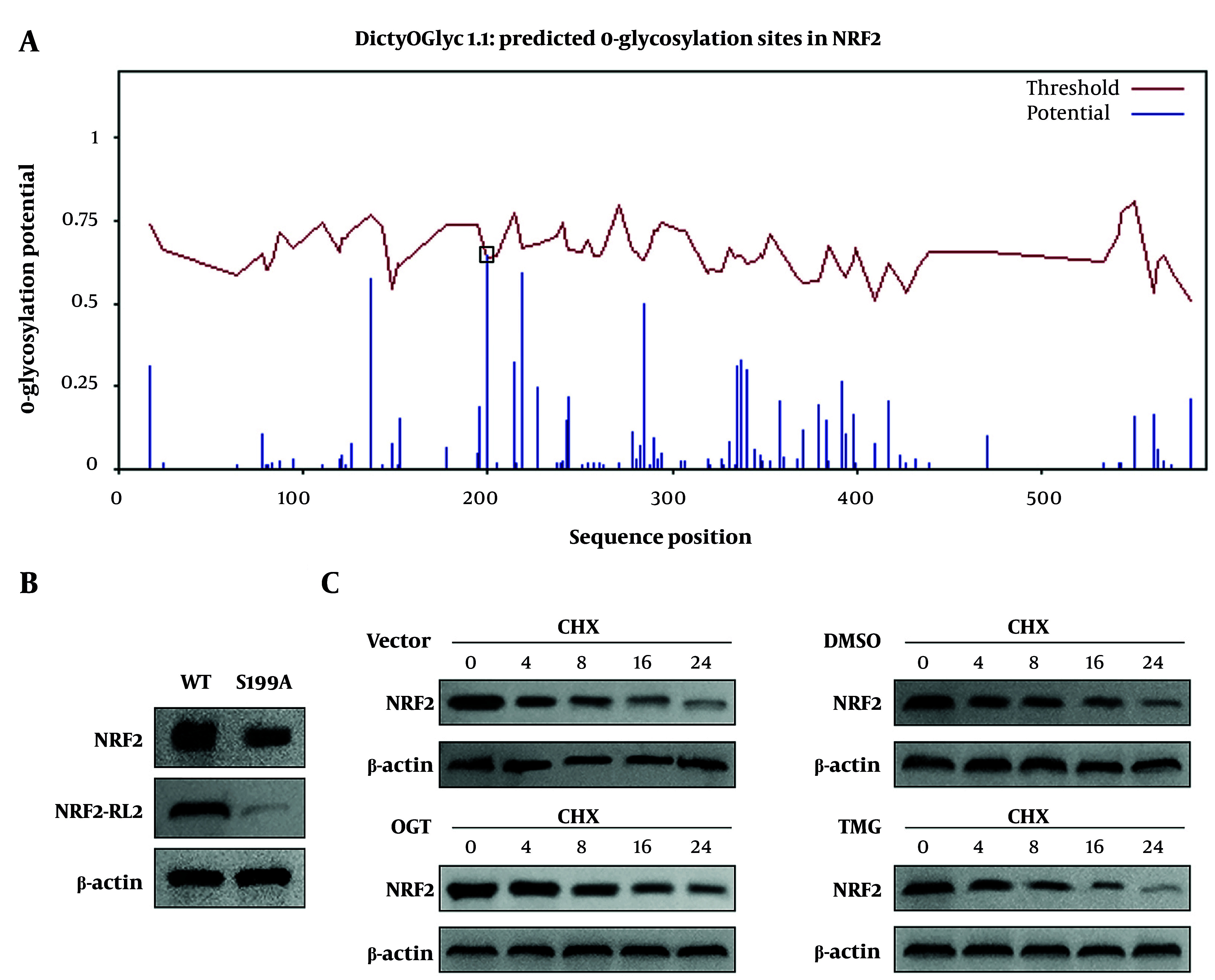
The regulation of OGT on O-GlcNAcylation and protein levels of Nrf2. A, the potential Nrf2 O-GlcNAcylation sites were predicted using the DictyOGlyc-1.1 database; S199: serine 199 residue of Nrf2, the highest-score predicted O-GlcNAcylation site. B, the total protein level and O-GlcNAc modification level of Nrf2 in WT (wild-type) Nrf2 and S199A (Nrf2 Ser199 alanine mutant) human hypertrophic scar fibroblasts (HHSFs) were detected by IP and western blot. C, Nrf2 protein stability in HHSFs was evaluated by western blot at 0, 4, 8, 16, and 24 h following 10 μM CHX (cycloheximide) treatment. DMSO, dimethyl sulfoxide, solvent control; TMG, O-(2-acetamido-2-deoxy-D-glucopyranosylidene)amino N-phenylcarbamate, a specific OGT inhibitor; β-actin was used as the internal reference for all western blot assays. n = 3 independent biological replicates.

To determine whether O-GlcNAcylation occurs on Nrf2, we investigated the effect of OGT on Nrf2 O-GlcNAcylation in HHSFs. Results showed that OGT overexpression upregulated the protein levels of OGT and Nrf2, and increased the O-GlcNAcylation of Nrf2 (Figure 3A). Co-IP indicated that there was an interaction between OGT and Nrf2 (Figure 3B). Moreover, immunofluorescence staining demonstrated co-localization between OGT and Nrf2 (Figure 3C). Subsequently, we predicted several potential O-GlcNAcylation sites on Nrf2, among which the S199 site exhibited the highest prediction score (Figure 4A). Therefore, we mutated this site to alanine (A) to confirm whether O-GlcNAcylation of Nrf2 occurs at this specific site. We found that the S199A mutation reduced both the protein levels and O-GlcNAcylation of Nrf2 (Figure 4B). Moreover, OGT overexpression enhanced the stability of Nrf2 protein, whereas TMG, an O-GlcNAcylation inhibitor, accelerated the degradation of Nrf2 (Figure 4C). Collectively, these findings demonstrated that OGT overexpression enhanced the stability of Nrf2 protein by increasing O-GlcNAcylation at the S199 site of Nrf2. To further elucidate the role of OGT in HS, we knocked down OGT expression in HHSFs. We observed that OGT knockdown suppressed cell viability, apoptosis, and pro-inflammatory cytokine release, while concurrently downregulating O-GlcNAcylation levels and the protein expression of both OGT and Nrf2 (Figure S2A-G). These findings confirm that OGT inhibition recapitulates the anti-fibrotic and anti-inflammatory effects of APS on HHSFs.

### 4.4. Nrf2 Overexpression Increases Cell Viability and Inflammation but Inhibits Apoptosis in APS-Treated Human Hypertrophic Scar Fibroblasts

**Figure 5.**
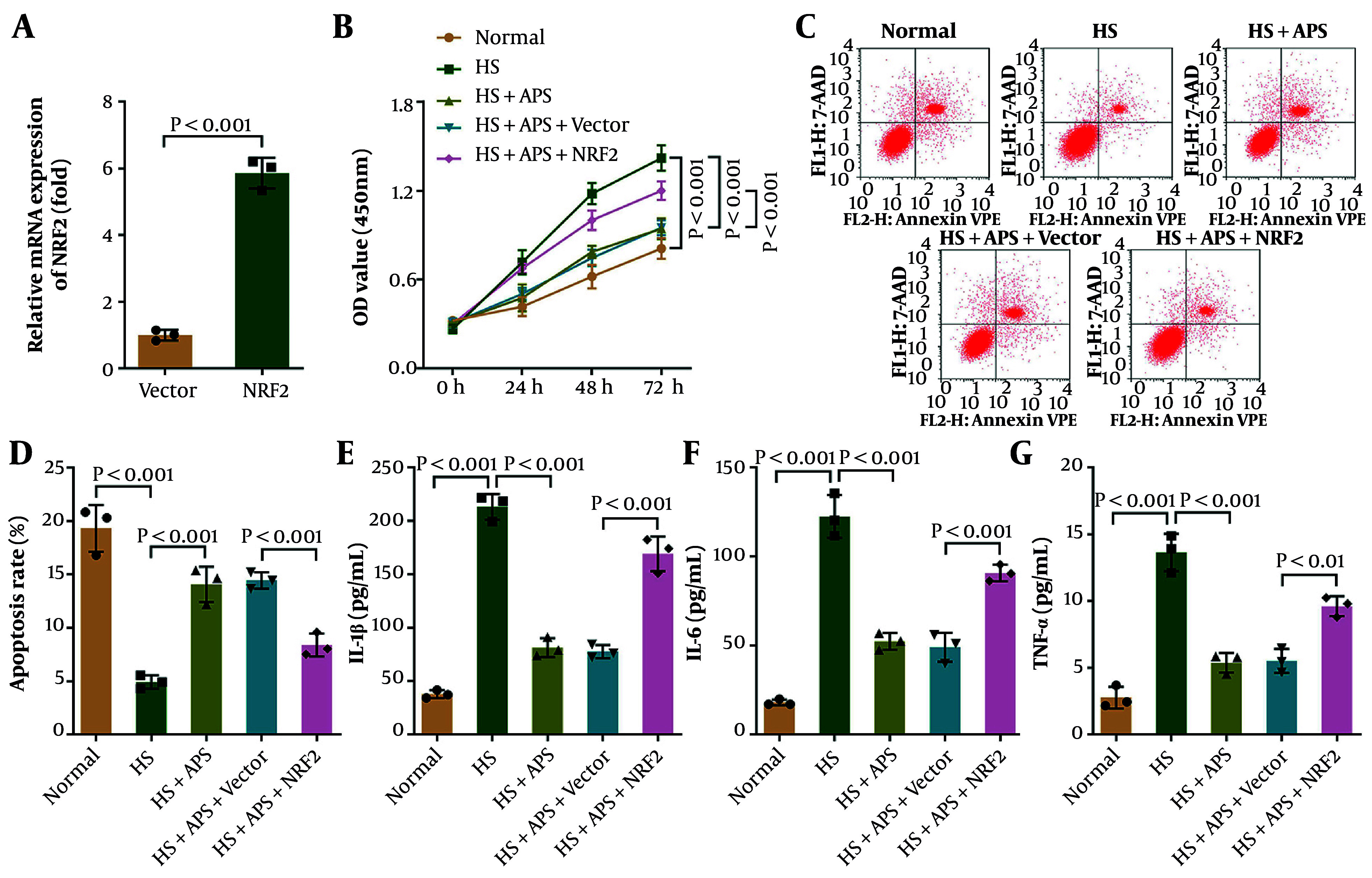
Nrf2 overexpression increased cell viability and inflammation but inhibited apoptosis in Astragalus polysaccharides (APS)-treated Human hypertrophic scar fibroblasts (HHSFs). A, Nrf2 mRNA expression in HHSFs was measured by qPCR (quantitative real-time polymerase chain reaction). B, cell viability of HHSFs and HSFs was detected using a CCK-8 kit. C and D, apoptosis of HHSFs and HSFs was evaluated by flow cytometry. E-G, the levels of IL-1β, IL-6, and TNF-α in HHSFs and HSFs were measured by ELISA. n = 3 independent biological replicates; P < 0.05 was considered statistically significant.

Finally, we investigated the role of Nrf2 in apoptosis and inflammation in HHSFs. Human hypertrophic scar fibroblasts were transfected with pcDNA3.1-Nrf2, and Nrf2 mRNA expression was significantly increased following transfection (Figure 5A). Results showed that cell viability in the HS group, decreased by APS, was partially restored by Nrf2 overexpression (Figure 5B). In contrast, Nrf2 overexpression significantly inhibited apoptosis facilitated by APS treatment in fibroblasts in the HS group (Figure 5C and D). Moreover, the reduced levels of IL-1β, IL-6, and TNF-α caused by APS treatment were partially increased following Nrf2 overexpression (Figure 5E-G). Furthermore, the levels of inflammatory cytokines in the S199A mutant group were significantly lower than those in the HS+APS+Nrf2 group, further demonstrating that OGT exerts its function by regulating Nrf2 O-GlcNAcylation at the S199 site (Figure S3A-C). In conclusion, our findings revealed that Nrf2 overexpression increased cell viability and inflammation but inhibited apoptosis in APS-treated HHSFs.

## 5. Discussion

In this study, we demonstrated that APS alleviated the formation of HS in a BLM-induced HS mouse model and inhibited inflammation while facilitating apoptosis of HHSFs. These findings are the first to demonstrate the therapeutic potential of APS for hypertrophic scar treatment. Astragalus polysaccharides is a major bioactive component extracted from the traditional Chinese herb Astragalus membranaceus and has a remarkable anti-inflammatory effect. In recent years, studies have demonstrated that APS participates in the regulation of inflammatory responses during wound healing. For example, another study (25) demonstrated that APS promotes wound healing and inhibits excessive inflammation at the late phase of wound healing in diabetic rats. Furthermore, Wen et al. (26) reported that an APS-loaded nanofibrous membrane demonstrated remarkable wound healing efficacy. The released APS exerted anti-inflammatory and pro-angiogenic effects, thereby significantly accelerating the healing process of articular wounds. Abnormal skin wound healing leads to the formation of HS, which is closely associated with dysregulated inflammatory responses during the wound healing process (27). Therefore, suppression of inflammation during wound healing is a key factor in preventing the formation of HS. However, the anti-inflammatory effects of APS on HS progression, as well as its therapeutic potential in modulating HS development, have not yet been investigated. In this study, we found that APS inhibited scar tissue formation and collagen deposition in HS mice and reduced the levels of pro-inflammatory cytokines IL-1β, IL-6, and TNF-α in vivo. Furthermore, APS suppressed cell viability and inflammatory responses in HHSFs while promoting their apoptosis. These results demonstrate that APS can inhibit HS progression and highlight its potential as a therapeutic agent for the prevention and treatment of HS. In addition, we found that APS inhibited O-GlcNAcylation of Nrf2 and reduced Nrf2 protein levels by downregulating OGT in the HS mouse model. As a central regulator of oxidative stress and inflammation, Nrf2 has emerged as a promising therapeutic target for various diseases. Its role in wound healing has been extensively investigated. Xiao et al. (6) revealed that Sangzhi alkaloids protect HUVECs against oxidative stress injury by activating the NRF2/HO-1/eNOS signaling pathway, thereby accelerating wound healing in diabetic conditions. Ganesh and Ramkuma (28) observed a defective Nrf2 response in diabetic mice with impaired wound closure due to excessive inflammation. Moreover, Wang et al. (29) demonstrated both in vitro and in vivo that targeting ferroptosis promotes wound healing through the activation of Nrf2. These results demonstrate that activation of Nrf2 is critical for promoting wound healing. However, the dual role of Nrf2 in tissue repair has been increasingly recognized in recent studies: moderate activation of Nrf2 promotes normal wound healing, while its persistent and excessive activation leads to abnormal fibroblast proliferation and ECM accumulation in fibrotic diseases (30-32). For example, Nrf2 overexpression has been shown to enhance the pro-fibrotic activity of fibroblasts in idiopathic pulmonary fibrosis (33), which is consistent with our finding that Nrf2 is abnormally upregulated in HHSFs. However, it remains unclear whether excessive activation of Nrf2 contributes to the development of HS. Li et al. (8) were the first to investigate the role of Nrf2 in HS, demonstrating that adipose-derived mesenchymal stem cells inhibit the proliferation and migration of HSFs, potentially through the downregulation of Nrf2 expression in HSFs. However, the role of Nrf2 in HS remains poorly understood. We found that Nrf2 protein level was upregulated in HSFs, which was decreased following APS treatment. Nrf2 overexpression promoted cell viability and inflammation but suppressed apoptosis in APS-treated HSFs. This result is also in line with the report that Nrf2 inhibition can induce fibroblast apoptosis and reduce inflammatory responses in hepatic fibrosis (34), suggesting a conserved regulatory role of Nrf2 in fibroblast biological behavior across different fibrotic diseases. These results demonstrate that elevated expression of Nrf2 promotes the progression of HS, and targeting the inhibition of Nrf2 is crucial for the prevention and treatment of HS development. Moreover, APS regulated Nrf2 expression through OGT-mediated O-GlcNAcylation in HSFs. OGT is the key enzyme responsible for catalyzing the addition of GlcNAc moieties to serine or threonine residues of target proteins during O-GlcNAcylation (18). O-GlcNAcylation modulates protein stability by interfering with the ubiquitination-dependent proteasomal degradation pathway, thereby participating in the progression of various diseases. Several studies have reported that OGT regulates Nrf2 expression through O-GlcNAcylation, thereby participating in the progression of various diseases. For instance, Zhang et al. (35) found that OGT regulates O-GlcNAcylation of Nrf2 at the Ser103 residue, thereby enhancing Nrf2 stability and promoting malignant progression and cisplatin resistance in lung cancer. Yang et al. (36) demonstrated that OGT upregulates Nrf2 expression through increased O-GlcNAcylation, thereby reducing ferroptosis susceptibility and protecting against lung ischemia-reperfusion injury. In this study, we demonstrated for the first time that OGT enhances the stability of Nrf2 by increasing its O-GlcNAcylation, thereby promoting the progression of HS. Furthermore, we show that APS inhibit this process by targeting OGT-mediated Nrf2 O-GlcNAcylation, leading to increased apoptosis in HSFs. These findings provide novel insights into the molecular mechanisms underlying HS development and identify a potential therapeutic target for the treatment of HS. While our study highlights the critical role of the OGT/Nrf2 axis in HS pathogenesis, it is important to consider its relationship with established fibrotic pathways. For instance, TGF-β/Smad signaling is well recognized as a central driver of fibrosis in HS and other fibrotic disorders. TGF-β promotes fibroblast activation, extracellular matrix deposition, and myofibroblast differentiation primarily through Smad-dependent and independent mechanisms (37, 38). Emerging evidence suggests crosstalk between Nrf2 and TGF-β signaling. For instance, TGF-β can induce ROS production, which in turn activates Nrf2-mediated antioxidant responses, implying a potential feedback loop (39). Conversely, Nrf2 activation has been reported to antagonize TGF-β-induced fibrogenesis by reducing oxidative stress and inflammation (40). Therefore, it is plausible that APS, by suppressing OGT-mediated Nrf2 O-GlcNAcylation, may indirectly influence TGF-β/Smad activity, thereby exerting its anti-fibrotic effects. Future studies are warranted to dissect the intricate interplay between the OGT/Nrf2 axis and TGF-β/Smad signaling in HS, which could reveal additional therapeutic targets. Despite the valuable insights gained, this study has certain limitations. First, the BLM-induced skin fibrosis model, while widely used for HS research, primarily mimics chemical-induced fibrosis and cannot fully recapitulate the pathological features of human HS (e.g., trauma-induced abnormal repair and scar contracture). Second, APS was administered via intraperitoneal injection in animal experiments, which differs from clinical local administration (e.g., topical application) for HS. The bioavailability and local targeting of APS after local administration remain unevaluated, affecting translational potential. Future studies should employ trauma-induced HS models and optimize local delivery systems of APS to enhance clinical applicability. In conclusion, this study demonstrates that APS alleviates HS progression by inhibiting OGT-mediated O-GlcNAcylation of Nrf2, thereby promoting fibroblast apoptosis and attenuating inflammation. These findings establish the OGT/Nrf2/O-GlcNAcylation signaling axis as a critical driver of HS pathogenesis and identify it as a novel therapeutic target. More broadly, this work highlights the potential of targeting protein O-GlcNAcylation in fibrotic diseases and supports the translational development of APS as a promising candidate for clinical scar management, pending further validation in more clinically relevant models and optimized delivery strategies.

## Data Availability

The datasets supporting the conclusions of this article are available in the DictyOGlyc-1.1 database (https://services.healthtech.dtu.dk/services/DictyOGlyc-1.1/).
